# P2X7R is involved in the progression of atherosclerosis by promoting NLRP3 inflammasome activation

**DOI:** 10.3892/ijmm.2015.2129

**Published:** 2015-03-09

**Authors:** KUANG PENG, LUSHAN LIU, DANGHENG WEI, YUNCHENG LV, GANG WANG, WENHAO XIONG, XIAOQING WANG, AFRASYAB ALTAF, LILI WANG, DAN HE, HONGYAN WANG, PENG QU

**Affiliations:** 1 Department of Cardiology, The Second Affiliated Hospital of Dalian Medical University, Dalian, Liaoning 116023, P.R. China; 2 Department of Cardiology, The First Affiliated Hospital of University of South China, Hengyang, Hunan 421001, P.R. China; 3 Institute of Cardiovascular Disease, Key Laboratory for Arteriosclerology of Hunan Province, University of South China, Hengyang, Hunan 421001, P.R. China; 4 Medical College, University of South China, Hengyang, Hunan 421001, P.R. China

**Keywords:** protein kinase R, purinergic 2X7 receptor, nucleotide-binding oligomerization domain-like receptor protein 3 inflammasome, atherosclerosis

## Abstract

Purinergic 2X7 receptor (P2X7R) and nucleotide-binding oligomerization domain-like receptor protein 3 (NLRP3) are expressed in macrophages in atherosclerotic lesions. However, the mechanisms through which P2X7R participates in the inflammatory response in atherosclerosis remain largely unknown. The aim of the present study was to investigate the role of P2X7R in atherosclerosis and the mechanisms of action of the NLRP3 inflammasome following stimulation with oxidized low-density lipoprotein (oxLDL). We observed the expression and distribution of P2X7R in the atherosclerotic plaque in the coronary arteries from an autopsy specimen and in that of the aortic sinuses of apoE^−/−^ mice by immunohistochemistry and immunofluorescence staining. The specificity of short interfering RNA (siRNA) was used to suppress P2X7R and NLRP3 mRNA expression. RT-qPCR and western blot analysis were used to analyze mRNA and protein expression, respectively. Co-immunoprecipitation was used to examine the interaction between protein kinase R (PKR) phosphorylation and NLRP3. P2X7R and NLRP3 were expressed at high levels in the atherosclerotic plaque in the coronary arteries. Stimulation with oxLDL upregulated P2X7R, NLRP3 and interleukin (IL)-1β expression. P2X7R knockdown by siRNA suppressed NLRP3 inflammasome activation by inhibiting the PKR phosphorylation mediated by oxLDL. In the atherosclerotic lesions in the aortic sinuses of apoE^−/−^ mice, P2X7R expression was found at high levels. Moreover, P2X7R siRNA attenuated the development of atherosclerosis in the apoE^−/−^ mice. In conclusion, our results demonstrate that P2X7R plays a significant role in the development of atherosclerosis and regulates NLRP3 inflammasome activation by promoting PKR phosphorylation.

## Introduction

The purinergic 2X7 receptor (P2X7R) is significantly expressed in CD31^+^ endothelial cells and CD68^+^ macrophages in atherosclerotic lesions in human carotid arteries (1). P2X7R may also modulate the release of several cytokines known to promote atherosclerosis, including interleukin (IL)-1β, IL-6 and tumor necrosis factor α through immune cells (2). Although this evidence suggests that there is an association between P2X7R and the development of atherosclerosis, the mechanisms through which P2X7R promotes the production of mature IL-1β by macrophages by modulating the nucleotide-binding oligomerization domain-like receptor protein 3 (NLRP3) inflammasome remain unknown.

P2X7R has attracted the most attention out of all the purinergic receptor family members, as it is involved in the inflammatory response by modulating the production of cytokines (such as IL-1β) by immune cells (2). However, the caspase-1 hydrolysis-dependent proIL-1β maturation also relies on NLRP3 inflammasome activity (7,8).

NLRP3 inflammasomes are known as cytoplasmic pattern recognition receptors (PRRs) (3,4). NLRP3 interacts with the adaptor molecule ASC (also known as PYD and CARD domain containing PYCARD) and caspase-1 to form a large multiprotein complex known as the NLRP3 inflammasome. Pro-caspase-1 is then converted into active caspase-1, which hydrolyzes proIL-1β into active IL-1β (5). The NLRP3 inflammasome can be activated by a wide range of stimuli, including intracellular cholesterol crystals (6,7), uric acid crystals (8,9), extracellular amyloids (10,11), adenosine triphosphate (ATP) (9,12,13), silicon dioxide and aluminum salts (14,15). A number of extracellular substances activate the NLRP3 inflammasome through P2X7R located on the plasma membrane (16,17).

*In vitro* studies have demonstrated that cholesterol crystals can activate the NLRP3 inflammasome and promote the release of IL-1β by macrophages with the assistance of lipopolysaccharides (LPS) (7,8). This is mainly due to the fact that IL-1β production involves a complex process and requires two independent signals (18,19). The first signal induces IL-1β mRNA expression through the Toll-like receptor 4 (TLR4) pathway, which is required for proIL-1β synthesis. The second signal is the lysozyme-dependent NLRP3 inflammasome activation and active caspase-1 production (8). Unlike LPS, cholesterol crystals cannot activate TLR4 signaling. Therefore, cholesterol crystals can only induce IL-1β production with the assistance of LPS (7,8). However, to the best of our knowledge, at present, evidence proving the high level of LPS in the plasma of patients with coronary heart disease is insufficient.

Thus, in addition to cholesterol crystals, there may be other endogenous substances activating the NLRP3 inflammasome in patients with atherosclerosis, such as oxidized low-density lipoprotein (oxLDL). Unlike cholesterol crystals, oxLDL has a function similar to LPS (20). oxLDL can promote the release of IL-1β through direct TLR4 activation independent of LPS (21–25). Therefore, it is worth investigating whether extracellular oxLDL activates the NLRP3 inflammasome through the upregulation of P2X7R in macrophages in a manner simlilar to amyloid A and ATP.

In this study, we analyzed the expression and distribution of P2X7R and NLRP3 in human coronary plaque. In order to understand the role of P2X7R in modulating NLRP3 inflammasome activity, we investigated the mechanisms behind the P2X7R-mediated NLRP3 inflammasome activation induced by oxLDL *in vitro*. We also examined the role of P2X7R in the progression of atherosclerosis in apoE^−/−^ mice. This study was approved by the Research Ethics Committee of the Second Affiliated Hospital of Dalian Medical University and the Animal Care and Use Committee of Dalian Medical University, Dalian, China.

## Materials and methods

### Antibodies and reagents

The anti-IL-1β and anti-NLRP3 antibodies used in the experiments in the present study were purchased from Proteintech Group (Chicago, IL, USA). Anti-caspase-1 and anti-CD68 antibodies were purchased from Wuhan Boster Biological Technology, Ltd. (Wuhan, China). Anti-P2X7 antibody was from Beijing Bioss Biosynthesis Biotechnology Co. Ltd. (Beijing, China). Anti-protein kinase R (PKR) (phospho T446) antibody was from Abcam (Cambridge, MA, USA). Short interfering RNA (siRNA) and negative control (NC) siRNA were purchased from Guangzhou RiboBio Co., Ltd. (Guangzhou, China).

### Cell preparation

THP-1, a human monocyte cell line, was obtained from the American Type Culture Collection (ATCC, Manassas, VA, USA). The cells were incubated in RPMI-1640 with 10% (v/v) heat-inactivated fetal bovine serum (FBS; Thermo Scientific HyClone, Logan, UT, USA), 100 U/ml penicillin and 100 g/ml streptomycin (Invitrogen/Life Technologies, Carlsbad, CA, USA). Phorbol 12-myristate 13-acetate (PMA; Sigma-Aldrich, St. Louis, MO, USA) was used to induce the differentiation of the THP-1 cells into macrophages and the cells were then cultured at 37°C in a humidified atmosphere of 5% CO_2_ in room air.

### Human coronary artery biopsy preparation

The paraffin section of the right coronary artery and the anterior descending branch of the left coronary artery from an autopsy specimen were provided by the Department of Pathology, University of South China, Hengyang, China.

### LDL isolation and modification

Native human LDL (nLDL) was isolated from plasma by ultracentrifugation. The density of the plasma was adjusted to 1.2 g/ml by the addition of solid potassium bromide (KBr). Following ultracentrifugation at 41,500 × g for 4 h at 4°C, very low-density lipoprotein was removed, 3.0 ml of the lower layer was transferred to another tube, 2 ml KBr-NaCl (d=1.18 g/ml) was added and the samples were gently mixed. The tubes were ultracentrifuged at 41,500 × g for 4 h at 4°C, and the LDL fraction was removed. The LDL fraction was dialyzed against 0.5 mM NaCl, pH 7.4, for 24 h at 4°C to remove EDTA. LDL was oxidized using CuSO_4_ as previously described (26).

### Histological analysis

Frozen cross-sections (8 *μ*m thick) of the aortic sinus of apoE^−/−^ mice and paraffin-embedded cross-sections of human coronary artery (5 *μ*m thick) were stained with primary antibodies (P2X7R, CD68 and NLRP3) and visualized with the appropriate secondary antibodies [from the SABC-POD Rabbit IgG IHC kit (SA1022); Wuhan Boster Biological Technology, Ltd.; goat anti-rabbit IgG, Cy3 conjugated (CW0159) and FITC conjugated (CW0114) secondary antibodies for fluorescence were from Cwbiotech, Beijing, China]. Conventional hematoxylin and eosin (H&E) staining of the pathological sections was performed. Oil Red O (kit provided by Nanjing Jiancheng Bioengineering Institute, Nanjing, China) was manipulated with specification for observing the cholesterol ester content in lipid-burdened cells and the frozen animal aortic sections. Section images were captured using Image-Pro Plus 6.0 software (Media Cybernetics, Inc., Rockville, MD, USA) and analyzed using Adobe Photoshop CS5 software (Adobe Systems Inc., San Jose, CA, USA).

### Mice

Male apoE knockout (apoE^−/−^) mice (6 weeks old with a C57BL/6 background) were supplied by Charles River Laboratories Inc. (Wilmington, MA, USA). For acclimation, the mice were fed a rodent chow diet for 1 week before being fed a high-cholesterol diet for 2, 4, 8 and 12 weeks (2% w/w cholesterol, 10% w/w lard). Mice in the control group were fed the rodent chow diet for 1 week. The hearts were collected and fixed by perfusion *in situ* with 10% neutralized formalin. The fixed hearts were then placed in a freezer at −20°C for use as frozen serial sections.

### Enzyme-linked immunosorbent assay (ELISA) for determining the levels of pro-inflammatory cytokines and oxLDL

Cytokine levels in the cell supernatant and mouse plasma were measured using ELISA kits (Neobioscience Technology Co., Ltd., Beijing, China). ELISA kits for mouse plasma oxLDL levels were from Cusabio Biotech Co., Ltd. (Wuhan, China).

### Reverse transcription-quantitative PCR (RT-qPCR)

Total RNA was extracted from the cells using the E.Z.N.A.^®^ HP Total RNA kit (Omega Bio-Tek, Inc., Norcross, GA, USA) following the instructions provided by the manufacturer. The RNA samples were converted into cDNA using the RevertAid First Strand cDNA Synthesis kit (Thermo Scientific Fermentas, Waltham, MA, USA). Quantitative PCR was carried out using the following primers: GAPDH sense, 5′-ATGACATCAAGAGGTGGTG-3′ and antisense, 5′-CATACCAGGAAATGAGCTTG-3′, annealing 60°C; NLRP3 sense, 5′-GTGTTTCGAAATCCCACTGTG-3′ and antisense, 5′-TCTGCTTCTCACGTACTTTCTG-3′, annealing 60°C and 57°C; P2X7R sense, 5′-GAACAATATCGACTTCCCCGG-3′ and antisense, 5′-TTATCGCCTGTTTCTCGGAAG3′, annealing 60°C and 55°C; IL-1β sense, 5′-CCTCCATTGATCATCTGTCTCTG3′ and antisense, 5′-GCTTGGATGTTTTAGAGGTTTCAG-3′, annealing 58°C.

### Western blot analysis and immunoprecipitation

The cells were suspended in the appropriate lysis buffer. Following centrifugation (10,000 rpm for 10 min), the protein content in the supernatants was quantified using the BCA protein assay kit (Beijing ComWin Biotech Co., Ltd., Beijing, China). The boiling denatured proteins were segregated by sodium dodecyl sulfate-polyacrylamide gel electrophoresis (SDS-PAGE) and transferred onto PVDF membranes (Millipore, Billerica, MA, USA) at 4°C. The membranes carrying proteins were incubated with primary antibody and detected with the appropriate secondary polyclonal antibody by BeyoECL Plus (Beyotime Institute of Biotechnology, Jiangsu, China). Antibodies against PKR and NLRP3 were used to precipitate proteins from cell lysis in the presence of 20 *μ*l protein A/G beads (Santa Cruz Biotechnology, Santa Cruz, CA, USA) overnight at 4°C. Protein complexes were washed 4 times with lysis buffer, and then incubated at 95°C for 5 min and resolved by western blot analysis.

### siRNA knockdown and transfection of cells and injection of siRNA into mice

The THP-1 macrophages were transfected with NLRP3 siRNA, P2X7R siRNA, or the negative control (NC) siRNA at 50 nM using the HiPerFect Transfection Reagent (Qiagen, Hilden, Germany). In the animal experiments, a lentivirus carrying P2X7R siRNA (GeneChem, Co., Ltd., Shanghai, China) was intravenously injected into tail veins of the apoE^−/−^ mice once every 2 weeks. The apoE^−/−^ mice in the negative control (NC) group were injected with a lentiviral vector carrying scrambled siRNA. Mice not injected with siRNA were used as the controls.

### Statistical analysis

Data are presented as the means ± standard deviation (SD). An unpaired two-tailed Student’s t-test was used for comparisons between 2 groups. Statistical analysis for 3 or more groups was performed using one-way ANOVA with the Tukey’s post-hoc test. A value of P<0.05 was considered to indicate a statistically significant differencde. Statistical analysis was performed using IBM SPSS statistical software (version 21.0).

## Results

### P2X7R and NLRP3 expression levels in atherosclerotic lesions in human coronary arteries

**Figure 1:**
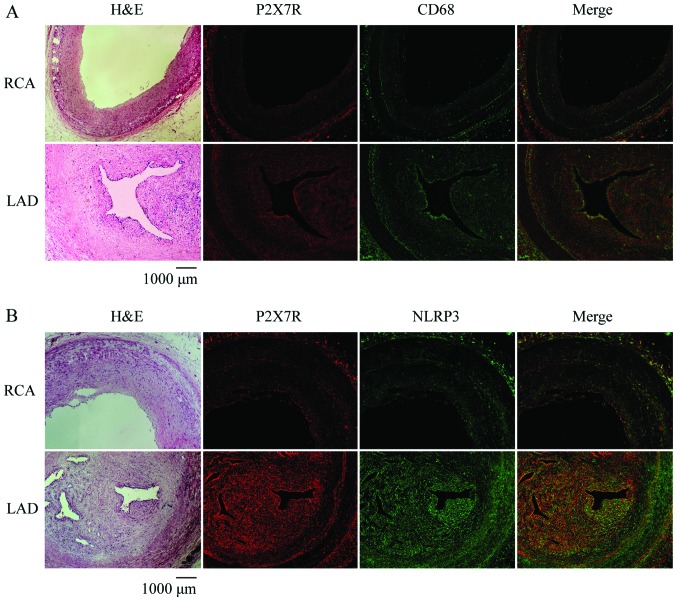
Purinergic 2X7 receptor (P2X7R) and nucleotide-binding oligomerization domain-like receptor protein 3 (NLRP3) expression and distribution in human atherosclerotic lesions. (A) P2X7R (red) and macrophage marker CD68 (green) expression. (B) P2X7R (red) and NLRP3 (green) expression. RCA, right coronary artery; LAD, anterior descending branch of left coronary artery; H&E, hematoxylin and eosin.

The morphological characteristics of the left anterior descending coronary artery and right coronary artery from the autopsy specimen was observed by H&E staining. The left anterior descending artery showed apparent eccentric stenosis with large amounts of foam cells compared to the right coronary artery (Fig. 1). Immunofluorescence staining for P2X7R, NLRP3 and the macrophage marker, CD68, using specific antibodies revealed the co-expression of P2X7R and NLRP3 in the atherosclerotic plaques (Fig. 1B) with large numbers of macrophages (Fig. 1A). This suggests that the high expression of P2X7R and NLRP3 in macrophages may be involved in the progression of atherosclerosis.

### Increased P2X7R expression in atherosclerotic lesions in apoE^−/−^ mice

**Figure 2:**
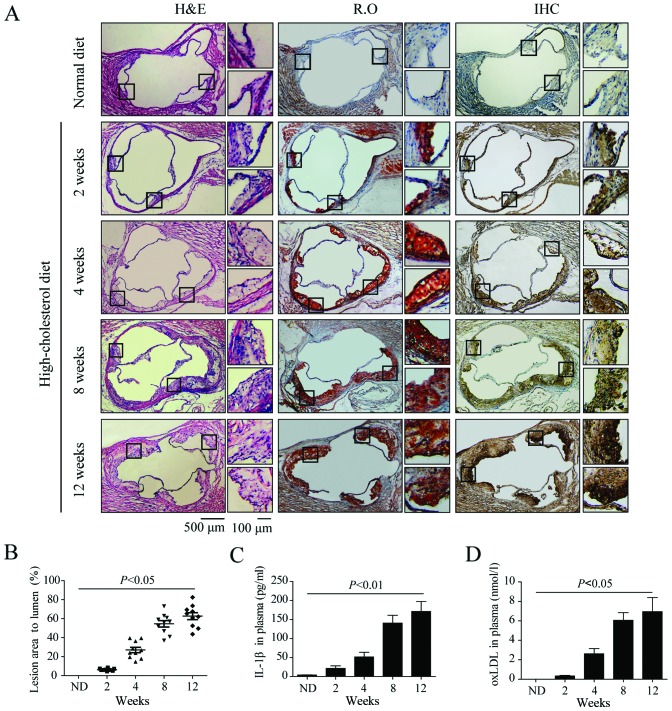
Purinergic 2X7 receptor (P2X7R) expression in atherosclerotic lesions in apoE^−/−^ mice fed a high-cholesterol diet for 2, 4, 8 and 12 weeks. (A) High P2X7R expression in lesions along with the development of atherosclerotic lesions in apoE^−/−^ mice fed a high-cholesterol diet for 2, 4, 8 and 12 weeks. H&E staining for aortic sinus lesions is shown in the 5 images on the left panel. Oil Red O staining for foam cells of atherosclerotic lesions is shown in the middle panel. Immunohistochemical analysis for the expression of P2X7R in the lesions is shown on the right panel. The black boxes in the images show the atherosclerotic predilection sites and the lesions. H&E, hematoxylin and eosin staining; RO, Oil Red O staining; IHC, immunohistochemistry. (B) Atherosclerotic lesion areas in the different groups of apoE^−/−^ mice (n=10). (C) Plasma interleukin-1β (IL-1β) concentration at different time points in apoE^−/−^ mice (n=10). (D) Plasma oxidized low-density lipoprotein (oxLDL) concentration at different time points in apoE^−/−^ mice (n=10). (C) Plasma IL-1β concentration at different time points in apoE^−/−^ mice (n=10). (D) Plasma oxidized low-density lipoprotein (oxLDL) concentration at different time points in apoE^−/−^ mice (n=10).

In this study, lesion size was determined by the percentage of atherosclerotic lesions compared to the entire cross-sectional vessel lumen area stained with H&E. There was only a small amount of foam cells in the aortic sinuses of apoE^−/−^ mice fed a high-cholesterol diet for 2 weeks, but P2X7R expression in the atherosclerotic lesions was significantly increased (Fig. 2A) compared with the control group (on a chow diet for 1 week). After 4 weeks on a high-cholesterol diet, more foam cells accumulated on the arterial wall with larger lesion areas compared to those at 2 weeks (Fig. 2A and B). Oil Red O staining and immunohistochemistry revealed that P2X7R was present in the foam cell-rich areas (Fig. 2A). Atherosclerotic plaque formed in the aortic sinuses of the apoE^−/−^ mice after 8 and 12 weeks on a high-cholesterol diet, and the lesion were significantly elevated toward the intimal surface with cholesterol crystals. There were many newly formed foam cells which hade accumulated in the lesions close to the lumen. P2X7R was highly expressed in the atherosclerotic plaques, particularly in the newly formed foam cells (Fig. 2A). ELISA revealed that the plasma IL-1β and oxLDL levels in the apoE^−/−^ mice fed a high-cholesterol diet for 4, 8 and 12 weeks were significantly higher than those of the mice in the control group (Fig. 2C and D).

### Effect of P2X7R on the formation of atherosclerotic lesions in apoE^−/−^ mice

**Figure 3:**
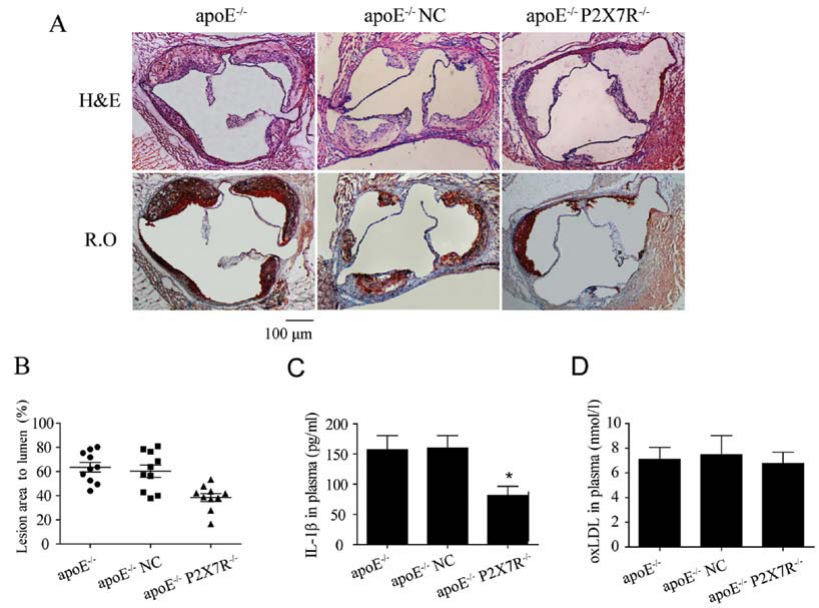
Effects of purinergic 2X7 receptor (P2X7R) on aortic atherosclerosis in apoE^−/−^ mice. (A) Effect of P2X7R knockdown on aortic atherosclerosis in apoE^−/−^ mice. (B) Atherosclerotic lesion areas in each group (n=10). (C) Plasma interleukin-1β (IL-1β) levels in each group (n=10). (D) Plasma oxidized low-density lipoprotein (oxLDL) levels in each group (n=10). H&E, hematoxylin and eosin; R.O, Oil Red O.

To suppress the function of P2X7R, a lentiviral vector carrying P2X7R siRNA was delivered to the apoE^−/−^ mice by a tail vein injection. The apoE^−/−^ mice in the negative control group were injected with a lentiviral vector carrying scrambled siRNA and the mice in the control group were not injected with siRNA. After 12 weeks on a high-cholesterol diet, the aortic sinus plaque areas in the apoE^−/−^ mice injected with P2X7R siRNA (apoE^−/−^ P2X7R^−/−^) were significantly smaller than those in the mice in the other 2 groups (Fig. 3A and B). More importantly, in addition to many foam cells, the lesions in the apoE^−/−^ mice and apoE^−/−^ NC mice exhibited significant extracellular lipid accumulation, necrotic tissue and some cholesterol crystals (Fig. 3A), indicating the formation of atherosclerotic plaque in the aortic sinuses. However, the aortic sinus lesions in the apoE^−/−^ P2X7R^−/−^ mice were mainly formed by foam cell accumulation, which is consistent with the early pathological characteristics of fatty streaks (Fig. 3A). However, a significantly delayed progression of atherosclerosis was observed in the apoE^−/−^ P2X7R^−/−^ mice (Fig. 3A and B).

Additionally, the plasma oxLDL levels were the same in each group, which indicates that the P2X7R knockdown did not affect the oxLDL plasma concentrations (Fig. 3D). However, the IL-1β levels in the plasma of apoE^−/−^ P2X7R^−/−^ mice were lower than those observed in the other 2 groups (Fig. 3C). This suggests that the development of atherosclerosis mediated by P2X7R in mice may be associated with the modulation of IL-1β activation and release.

### P2X7R affects foam cell formation

**Figure 4:**
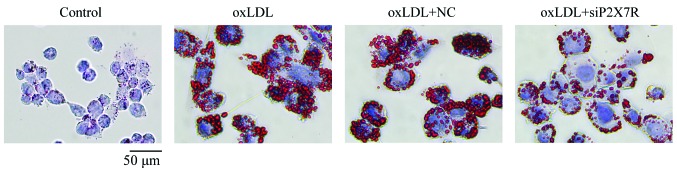
Oil Red O staining of macrophages. The oxidized low-density lipoprotein (oxLDL) and oxLDL + negative control (NC) groups had high lipid-loaded macrophage and foam cell formation levels. Purinergic 2X7 receptor (P2X7R) knockdown by short interfering RNA (siRNA) significantly reduced the formation of lipid-loaded macrophages.

Oil Red O staining revealed that many lipid droplets had accumulated in the THP-1 macrophages after 24 h of incubation with 100 *μ*g/ml oxLDL (Table I and Fig. 4). Additionally, the cytoplasmic cholesterol ester content accounted for >50% of the total cholesterol. This indicates foam cell formation. There were significantly fewer cholesterol esters (Table I) and lipid droplets (Fig. 4) in the P2X7R siRNA-treated macrophages compared to the negative controls, suggesting that P2X7R affects lipid loading in macrophages and foam cell formation.

### Regulatory role of oxLDL in the induction of P2X7R, NLRP3 and IL-1β expression in macrophages

**Figure 5:**
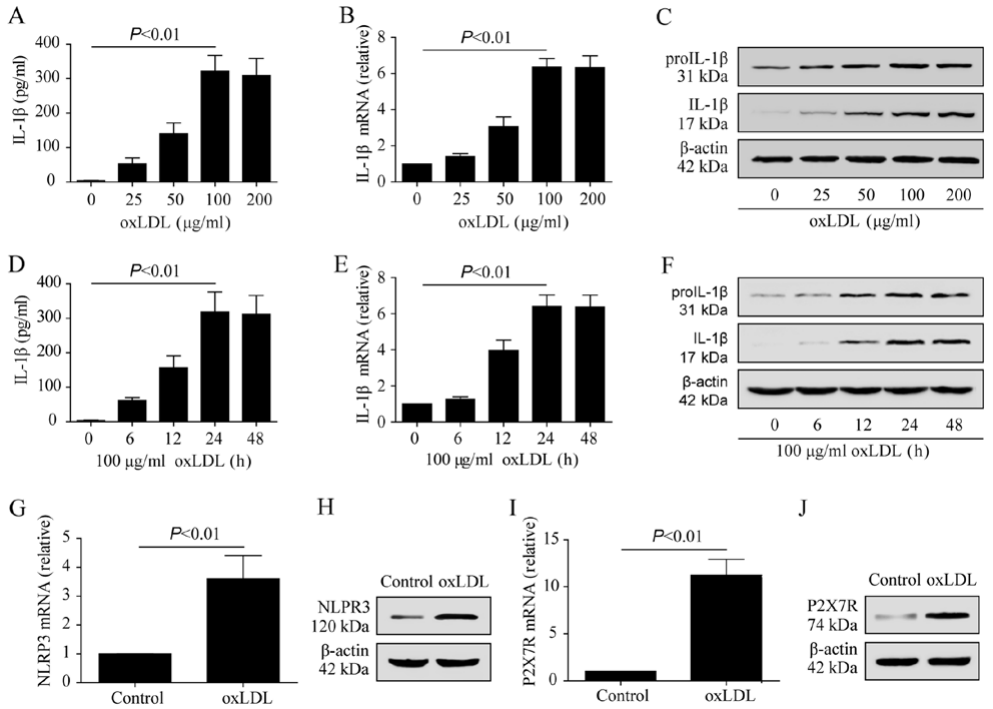
Oxidized low-density lipoprotein (oxLDL) promotes THP-1 macrophage production and the release of interleukin-1β (IL-1β) by activating the purinergic 2X7 receptor (P2X7R)/nucleotide-binding oligomerization domain-like receptor protein 3 (NLRP3) pathway. (A) IL-1β concentration in medium after THP-1 cells were stimulated with different concentrations of oxLDL. (B) IL-1β mRNA expression after THP-1 cells were stimulated with different concentrations of oxLDL. (C) IL-1β expression after THP-1 cells were stimulated with different concentrations of oxLDL. (D) IL-1β concentration in medium at different time points after THP-1 cells were stimulated with 100 *μ*g/ml oxLDL. (E) IL-1β mRNA expression at different time points after THP-1 cells were stimulated with 100 *μ*g/ml oxLDL. (F) IL-1β expression at different time points after THP-1 cells were stimulated with 100 *μ*g/ml oxLDL. (G and H) Effect of oxLDL on NLRP3 expression in THP-1 macrophages. (I and J) Effect of oxLDL on P2X7R expression in THP-1 macrophages.

To demonstrate that oxLDL upregulates P2X7R and NLRP3 in THP-1 macrophages and promotes the secretion of IL-1β, the THP-1 macrophages were stimulated with 25, 50, 100 and 200 *μ*g/ml oxLDL for 24 h. The IL-1β concentrations were significantly higher in the oxLDL-treated groups compared to the control group (Fig. 5A). The cytoplasmic proIL-1β mRNA and protein levels, as well as the mature IL-1β protein levels, were increased in the THP-1 macrophages treated with oxLDL (Fig. 5B and C). A concentration of 100 *μ*g/ml of oxLDL was selected to treat the THP-1 macrophages for 6, 12, 24 and 48 h to examine the IL-1β expression and secretion. Following treatment with 100 *μ*g/ml oxLDL for 24 h, the expression of mature IL-1β and proIL-1β increased compared to the control group (Fig. 5E and F). IL-1β medium concentration was also significantly increased (Fig. 5D). These results indicated that oxLDL upregulated IL-1β expression in THP-1 macrophages and promoted proIL-1β hydrolysis to activate IL-1β. oxLDL also upregulated the the mRNA and protein expression levels of P2X7R and NLRP3 in the THP-1 macrophages (Fig. 5G–J).

### Regulatory role of P2X7R and the NLRP3 inflammasome in the production of IL-1β in macrophages

**Figure 6:**
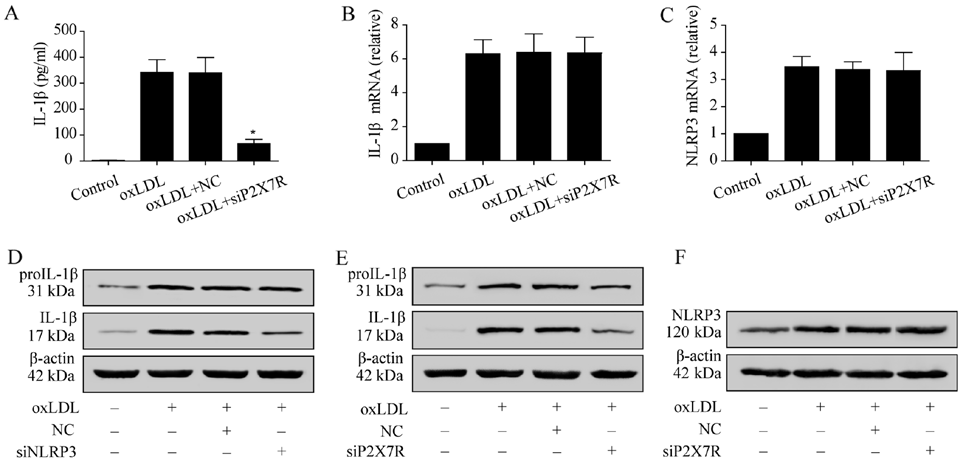
Effect of purinergic 2X7 receptor (P2X7R) on THP-1 macrophage production and secretion of interleukin-1β (IL-1β). (A) Effect of P2X7R on IL-1β concentration in medium secreted by THP-1 macrophages. P2X7R knockdown by short interfering RNA (siRNA) significantly reduced the IL-1β concentration in medium compared with the control group (P<0.01). (B) Effect of P2X7R on IL-1β mRNA expression in THP-1 macrophages. (C) Effect of P2X7R on THP-1 macrophage nucleotide-binding oligomerization domain-like receptor protein 3 (NLRP3) mRNA expression in THP-1 macrophages. (D) oxLDL promoted production of IL-1β by THP-1 macrophages, and this was related to the NLRP3 inflammasome activation. (E) Effect of P2X7R on proIL-1β expression in and mature IL-1β production by THP-1 macrophages. (F) Effect of P2X7R on NLRP3 expression in THP-1 macrophages. NC, negative control.

The IL-1β mRNA and proIL-1β protein expression levels in the oxLDL-stimulated macrophages did not differ between the negative control group and the P2X7R siRNA-treated group (Fig. 6B and E). However, proIL-1β activity was significantly suppressed (Fig. 6E). The concentration of IL-1β in the medium was also reduced due to the suppression of P2X7R (Fig. 6A), since proIL-1β hydrolysis to mature IL-1β is controlled by NLRP3 inflammasome activation. After NLRP3 expression was suppressed using siRNA, mature IL-1β expression in the oxLDL-stimulated macrophages was significantly lower than that in the negative controls, while proIL-1β expression was unaltered (Fig. 6D). However, NLRP3 expression in the macrophages in which P2X7R was knocked down following oxLDL stimulation did not differ from that observed in the negative control group (Fig. 6C and F). These results indicate that, upon oxLDL stimulation, the expression of P2X7R in macrophages regulates NLRP3 inflammasome function, but not NLRP3 expression.

### P2X7R regulates NLRP3 inflammasome assembly by promoting PKR phosphorylation

**Figure 7:**
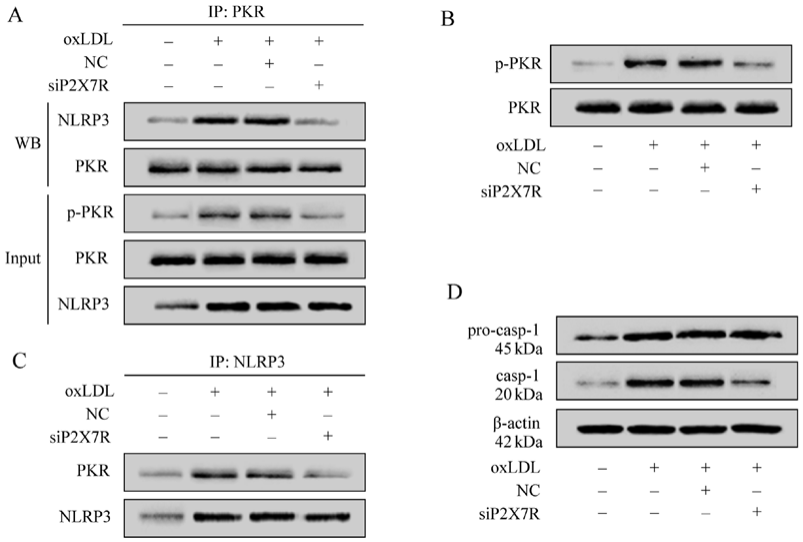
Purinergic 2X7 receptor (P2X7R) modulates nucleotide-binding oligomerization domain-like receptor protein 3 (NLRP3) inflammasome activity by upregulating PKR phosphorylation. (A) Co-immunoprecipitation assay to determine the interaction between NLRP3 and PKR using specific antibodies. (B) P2X7R regulation of PKR phosphorylation. (C) Co-immunoprecipitation assay to determine the interaction between PKR and NLRP3 using specific antibodies. (D) P2X7R activation of caspase-1. NC, Negative control.

To verify that P2X7R affects NLRP3 inflammasome activity by regulating PKR phosphorylation upon stimulation with oxLDL, we evaluated the phosphorylation level of PKR in the THP-1 macrophages using specific antibodies, and investigated the interaction between phosphorylated PKR and NLRP3 using a co-immunoprecipitation method. The results revealed that treatment with oxLDL promoted PKR phosphorylation, upregulated NLRP3 expression (Fig. 7A–C) and produced a large amount of active caspase-1 (Fig. 7D). The regulation of PKR phosphorylation by oxLDL was significantly attenuated when P2X7R was suppressed, which suggested that oxLDL promoted PKR phos-phorylation by upregulating P2X7R. To examine the interaction between PKR and NLRP3, we used a specific antibody to PKR in the co-immunoprecipitation assay, which revealed that a large amount of PKR formed a complex with NLRP3 after being phosphorylated by oxLDL (Fig. 7A and C). After P2X7R was suppressed by siRNA, the oxLDL-induced PKR phosphorylation was significantly decreased. Consequently, there was a reduced interaction between P2X7R and NLRP3 and a reduced caspase-1 activity (Fig. 7D). As the production of active caspase-1 signals NLRP3 inflammasome assembly and activation, these results indicate that oxLDL regulates NLRP3 inflammasome activity by upregulating P2X7R expression and promoting PKR phosphorylation.

## Discussion

P2X7R (27) and NLRP3 (28) are abundant in immune cells, such as mononuclear phagocytes, dendritic cells and T cells. A number of substances activate the NLRP3 inflammasome through the P2X7R pathway (29–31). Niemi *et al* (10) demonstrated that serum amyloid A (SAA) induced proIL-1β expression and activated the inflammasome, promoting macrophages to secrete mature IL-1β. The suppression of caspase-1 and P2X7R hampered the production of mature IL-1β triggered by SAA (10). Gicquel *et al* (12) found that the secretion of IL-1β by ATP-stimulated macrophages involved the P2X7R/NLRP3 inflammasome pathway.

In the present study, in the plaque of the left anterior descending coronary artery, P2X7R and NLRP3 were highly co-expressed in areas with macrophage accumulation (Fig. 1). This suggests that the high expression levels of P2X7R and NLRP3 in macrophages are involved in the progression of atherosclerosis. This is supported by Piscopiello *et al* (1), who observed high P2X7R expression in CD31^+^ endothelial cells and CD68^+^ macrophages in human carotid atherosclerotic lesions.

To further illustrate the role of P2X7R in the development of atherosclerosis, this study examined the expression of P2X7R in the arterial walls of apoE^−/−^ mice fed a high cholesterol diet. We demonstrated that, in the atherosclerotic lesions, the expression of P2X7R was high in the areas with foam cell accumulation (Fig. 2A and B). The plasma levels of IL-1β in mice with atherosclerosis were also significantly higher than those observed in the control group (Fig. 2C). However, P2X7R expression detected in human coronary atherosclerotic plaque is not sufficient to prove that P2XR7 is involved in the development of atherosclerosis. It was thus necessary to observe the effects of the knockdown of P2X7R on atherosclerosis. *In vivo*, the knockdown of P2X7R in apoE^−/−^ mice significantly delayed the development of atherosclerosis (Fig. 3A and B) with lower plasma levels of IL-1β (Fig. 3C). In the apoE^−/−^ mice injected with P2XR7 siRNA, the atherosclerotic lesion area in the aortic root was smaller compared to that in the negative control group, but foam cells were the main component in lesions where cholesterol crystals and necrotic material were not found.

These findings indicate that P2X7R, by modulating IL-1β maturation, plays an important role in the development of atherosclerosis. This is also supported by the finding of a previous study demonstrating that apoE^−/−^ Casp^−/−^ mice had suppressed plaque areas and macrophage infiltration compared to apoE^−/−^ mice (32). In our animal experiments, P2X7R expression in foam cell-rich areas is particularly prominent; therefore, in this study, we also examined the effects of P2X7R expression on macrophage foam cell formation *in vitro*. After the cells were incubated with oxLDL for 24 h, macrophage foam cell formation was evident. P2X7R knockdown significantly delayed this formation (Table I and Fig. 4). Although no studies have suggested that P2X7R in macrophages directly mediates lipid phagocytosis, P2X7R has been shown to promote foam cell formation by modulating the NLRP3/IL-1β pathway (5,34).

Evidence from the present and previous studies suggests that, in atherosclerosis, oxLDL is the most likely candidate to upregulate P2X7R and activate the NLRP3 inflammasome. Firstly, the present study showed that oxLDL promoted macrophage foam cell formation, while macrophage foam cell formation was suppressed by P2X7R knockdown. Moreover, *in vivo*, P2X7R knockdown delayed the progression of atherosclerosis. Secondly, immunohistochemical studies have revealed that the NLRP3 inflammasome is activated in plaque macrophages (19), while oxLDL modulates NLRP3 inflammasome activity in mouse macrophages and activates caspase-1 (34,35). Thirdly, a number of substances, such as amyloid protein A (10,11) and ATP (9,12,13) have been shown to activate the NLRP3 inflammasome by upregulating P2X7R expression. Finally, patients with coronary heart disease exhibit higher plasma oxLDL levels (36). Moreover, studies have shown that patients with coronary artery disease do not exhibit high plasma LPS concentrations, and that oxLDL differs from cholesterol crystals (7,8) in that it can activate TLR4 without LPS, while oxLDL itself also has a cholesterol component (21–25).

To verify our hypothesis, we incubated the THP-1 macrophages with oxLDL. oxLDL not only upregulated P2X7R, NLRP3 and proIL-1β expression, but also promoted the production and release of active IL-1β (Fig. 5A–J). Masters *et al* (20) found that oxLDL upregulated IL-1β mRNA expression and promoted the release of IL-1β by mouse bone marrow-derived macrophages. These conclusions are consistent with those of the present study. By knocking down the expression of P2X7R and NLRP3 in macrophages using specific siRNA, we demonstrated that the generation of mature IL-1β upon oxLDL stimulation was dependent on P2X7R and the NLRP3 inflammasome (Fig. 6D and E). Since proIL-1β maturation is directly controlled by the NLRP3 inflammasome under the effects of oxLDL, P2X7R either modulates NLRP3 expression or the NLRP3 inflammasome assembly and activation.

Our experiments revealed that, after the P2X7R knockdown, proIL-1β maturation was evidently inhibited, but oxLDL still significantly upregulated NLRP3 and proIL-1β expression. This indicates that, upon oxLDL stimulation, P2X7R in macrophages modulates proIL-1β maturation processes (Fig. 6A, B and E), but does not regulate NLRP3 and proIL-1β expression (Fig. 6B, C, E and F). Therefore, P2X7R is likely to affect NLRP3 inflammasome activity by regulating its assembly. In the study by Lu *et al* (37), it was demonstrated that phosphorylated PKR, under the effects of ATP, is essential for NLRP3 inflammasome assembly and activation. It should be noted that, although ATP is considered a classic ligand to P2X7R, these studies did not investigate the regulatory effects of P2X7R on NLRP3 inflammasome activity. In this study, we demonstrated that, upon oxLDL stimulation, P2X7R knockdown reduced PKR phosphorylation and the amount of NLRP3 combined with PKR (Fig. 7A and B). NLRP3 inflammasome-mediated caspase-1 (Fig. 7D) and mature IL-1β levels were also significantly reduced (Fig. 6D). Therefore, P2X7R may regulate NLRP3 inflammasome activity by regulating NLRP3 inflammasome assembly rather than affecting its expression.

In the development of atherosclerosis, cholesterol may activate the NLRP3 inflammasome in two ways. One includes cholesterol crystal formation following endocytosis through receptors related to lipid metabolism, such as CD36 (21,38,39). These cholesterol crystals activate the NLRP3 inflammasome through the cathepsin B-dependent destabilizing lysosome (7,8). The second way is by upregulating macrophage NLRP3 and IL-1β mRNA and protein expression in the form of oxLDL through the TLR4/NF-κB pathway (33). At the same time, oxLDL initiates phosphorylated PKR interaction with NLRP3 by upregulating P2X7 receptors, and thus promotes NLRP3 inflammasome assembly and activation.

In conclusion, the present study demonstrates that P2X7R and NLRP3 are expressed at hight levels in foam cell-rich human coronary atherosclerotic lesion areas, and that P2X7R is involved in the progression of atherosclerosis *in vivo* and *in vitro*. Upon oxLDL stimulation, P2X7R upregulation modulates NLRP3 inflammasome activation by enhancing PKR phosphorylation, which promotes the production of mature IL-1β by macrophages. This is possibly one of the mechanisms through which P2X7R participates in the progression and development of atherosclerosis.

## Figures and Tables

**Table I:** Cholesterol and cholesterol esters in THP-1 macrophages (mg/g, n=3).

Group	TC	FC	CE	CE/TC (%)
Control	194.2±16.7	143.4±12.1	51.1±4.3	26.3
oxLDL	464.8±13.5	188.2±17.7	276.3±15.8^a^	59.4
oxLDL + NC	459.1±12.2	175.5±9.3	284.6±19.2^a^	61.8
oxLDL + siP2X7R	282.4±19.1	174.7±7.3	107.8±11.7^b^	37.9

a Compared with the control group, P<0.01;

b compared with oxLDL + NC group, P<0.05. TC, total cholesterol; FC, free cholesterol; CE, cholesterol ester; siP2X7R, purinergic 2X7 receptor siRNA; NC, negative control.

## References

[1] Piscopiello M, Sessa M, Anzalone N (2013). P2X7 receptor is expressed in human vessels and might play a role in atherosclerosis. Int J Cardiol.

[2] Mehta N, Kaur M, Singh M (2014). Purinergic receptor P2X7: A novel target for anti-inflammatory therapy. Bioorg Med Chem.

[3] Wang LL, Wang HY, Qu P (2012). Role and relationships of pattern-recognition receptors in atherosclerosis (In Chinese). Chin J Arterioscler.

[4] Jia Y, Zhou L, Li XH (2014). Advance in the study of nod-like receptor protein-3 inflammasome and atherosclerosis (In Chinese). Chin J Arterioscler.

[5] Yang CS, Shin DM, Jo EK (2012). The role of NLR-related protein 3 inflammasome in host defense and inflammatory diseases. Int Neurourol J.

[6] Grebe A, Latz E (2013). Cholesterol crystals and inflammation. Curr Rheumatol Rep.

[7] Duewell P, Kono H, Rayner KJ (2010). NLRP3 inflammasomes are required for atherogenesis and activated by cholesterol crystals. Nature.

[8] Rajamäki K, Lappalainen J, Oörni K (2010). Cholesterol crystals activate the NLRP3 inflammasome in human macrophages: a novel link between cholesterol metabolism and inflammation. PLoS One.

[9] Riteau N, Baron L, Villeret B (2012). ATP release and purinergic signaling: a common pathway for particle-mediated inflammasome activation. Cell Death Dis.

[10] Niemi K, Teirilä L, Lappalainen J (2011). Serum amyloid A activates the NLRP3 inflammasome via P2X7 receptor and a cathepsin B-sensitive pathway. J Immunol.

[11] Eklund KK, Niemi K, Kovanen PT (2012). Immune functions of serum amyloid A. Crit Rev Immunol.

[12] Gicquel T, Victoni T, Fautrel A (2014). Involvement of purinergic receptors and NOD-like receptor-family protein 3-inflammasome pathway in the adenosine triphosphate-induced cytokine release from macrophages. Clin Exp Pharmacol Physiol.

[13] Gombault A, Baron L, Couillin I (2013). ATP release and purinergic signaling in NLRP3 inflammasome activation. Front Immunol.

[14] Kuroda E, Ishii KJ, Uematsu S (2011). Silica crystals and aluminum salts regulate the production of prostaglandin in macrophages via NALP3 inflammasome-independent mechanisms. Immunity.

[15] Hornung V, Bauernfeind F, Halle A (2008). Silica crystals and aluminum salts activate the NALP3 inflammasome through phagosomal destabilization. Nat Immunol.

[16] Tschopp J, Schroder K (2010). NLRP3 inflammasome activation: The convergence of multiple signalling pathways on ROS production?. Nat Rev Immunol.

[17] Latz E, Xiao TS, Stutz A (2013). Activation and regulation of the inflammasomes. Nat Rev Immunol.

[18] Wen H, Miao EA, Ting JP (2013). Mechanisms of NOD-like receptor-associated inflammasome activation. Immunity.

[19] McGettrick AF, O’Neill LA (2013). How metabolism generates signals during innate immunity and inflammation. J Biol Chem.

[20] Masters SL, Dunne A, Subramanian SL (2010). Activation of the NLRP3 inflammasome by islet amyloid polypeptide provides a mechanism for enhanced IL-1β in type 2 diabetes. Nat Immunol.

[21] Chávez-Sánchez L, Garza-Reyes MG, Espinosa-Luna JE, Chávez-Rueda K, Legorreta-Haquet MV, Blanco-Favela F (2014). The role of TLR2, TLR4 and CD36 in macrophage activation and foam cell formation in response to oxLDL in humans. Hum Immunol.

[22] Björkbacka H, Kunjathoor VV, Moore KJ (2004). Reduced atherosclerosis in MyD88-null mice links elevated serum cholesterol levels to activation of innate immunity signaling pathways. Nat Med.

[23] West XZ, Malinin NL, Merkulova AA (2010). Oxidative stress induces angiogenesis by activating TLR2 with novel endogenous ligands. Nature.

[24] Miller YI, Viriyakosol S, Binder CJ, Feramisco JR, Kirkland TN, Witztum JL (2003). Minimally modified LDL binds to CD14, induces macrophage spreading via TLR4/MD-2, and inhibits phagocytosis of apoptotic cells. J Biol Chem.

[25] Mullick AE, Tobias PS, Curtiss LK (2005). Modulation of atherosclerosis in mice by Toll-like receptor 2. J Clin Invest.

[26] Chávez-Sánchez L, Chávez-Rueda K, Legorreta-Haquet MV (2010). The activation of CD14, TLR4, and TLR2 by mmLDL induces IL-1β, IL-6, and IL-10 secretion in human monocytes and macrophages. Lipids Health Dis.

[27] Wiley JS, Sluyter R, Gu BJ, Stokes L, Fuller SJ (2011). The human P2X7 receptor and its role in innate immunity. Tissue Antigens.

[28] Benetti E, Chiazza F, Patel NS, Collino M (2013). The NLRP3 Inflammasome as a novel player of the intercellular crosstalk in metabolic disorders. Mediators Inflamm.

[29] Solini A, Menini S, Rossi C (2013). The purinergic 2X7 receptor participates in renal inflammation and injury induced by high-fat diet: possible role of NLRP3 inflammasome activation. J Pathol.

[30] Hussen J, Düvel A, Koy M, Schuberth HJ (2012). Inflammasome activation in bovine monocytes by extracellular ATP does not require the purinergic receptor P2X7. Dev Comp Immunol.

[31] Dekali S, Divetain A, Kortulewski T (2013). Cell cooperation and role of the P2X7 receptor in pulmonary inflammation induced by nanoparticles. Nanotoxicology.

[32] Usui F, Shirasuna K, Kimura H (2012). Critical role of caspase-1 in vascular inflammation and development of atherosclerosis in Western diet-fed apolipoprotein E-deficient mice. Biochem Biophys Res Commun.

[33] Qiao Y, Wang P, Qi J, Zhang L, Gao C (2012). TLR-induced NF-κB activation regulates NLRP3 expression in murine macrophages. FEBS Lett.

[34] Matsuura E, Lopez LR, Shoenfeld Y, Ames PR (2012). β2-glycoprotein I and oxidative inflammation in early atherogenesis: a progression from innate to adaptive immunity?. Autoimmun Rev.

[35] Lin J, Shou X, Mao X (2013). Oxidized low density lipoprotein induced caspase-1 mediated pyroptotic cell death in macrophages: implication in lesion instability?. PLoS One.

[36] Ehara S, Ueda M, Naruko T (2001). Elevated levels of oxidized low density lipoprotein show a positive relationship with the severity of acute coronary syndromes. Circulation.

[37] Lu B, Nakamura T, Inouye K (2012). Novel role of PKR in inflammasome activation and HMGB1 release. Nature.

[38] Oury C (2014). CD36: linking lipids to the NLRP3 inflammasome, atherogenesis and atherothrombosis. Cell Mol Immunol.

[39] Sheedy FJ, Grebe A, Rayner KJ (2013). CD36 coordinates NLRP3 inflammasome activation by facilitating intracellular nucleation of soluble ligands into particulate ligands in sterile inflammation. Nat Immunol.

